# Burden of pneumocystis pneumonia in HIV-infected adults in sub-Saharan Africa: a systematic review and meta-analysis

**DOI:** 10.1186/s12879-016-1809-3

**Published:** 2016-09-09

**Authors:** Sean Wasserman, Mark E. Engel, Rulan Griesel, Marc Mendelson

**Affiliations:** 1 Division of Infectious Diseases and HIV Medicine, Department of Medicine, University of Cape Town, Cape Town, South Africa; 2 Department of Medicine, University of Cape Town, Cape Town, South Africa; 3 Division of Clinical Pharmacology, Department of Medicine, University of Cape Town, Cape Town, South Africa

**Keywords:** Pneumocystis pneumonia, HIV-associated opportunistic infection, Respiratory disease in HIV, PCP

## Abstract

**Background:**

Seroprevalence data and clinical studies in children suggest that the burden of pneumocystis pneumonia (PCP) in Africa may be underestimated. We performed a systematic review to determine the prevalence and attributable mortality of PCP amongst HIV-infected adults in sub-Saharan Africa.

**Methods:**

We searched Pubmed, Web of Science, Africa-Wide: NiPAD and CINAHL, from Jan 1 1995 to June 1 2015, for studies that reported the prevalence, mortality or case fatality of PCP in HIV-infected adults living in sub-Saharan African countries. Prevalence data from individual studies were combined by random-effects meta-analysis according to the Mantel-Haenszel method. Data were stratified by clinical setting, diagnostic method, and study year.

**Results:**

We included 48 unique study populations comprising 6884 individuals from 18 countries in sub-Saharan Africa. The pooled prevalence of PCP among 6018 patients from all clinical settings was 15 · 4 % (95 % CI 12 · 9–18 · 0), and was highest amongst inpatients, 22 · 4 % (95 % CI 17 · 2–27 · 7). More cases were identified by bronchoalveolar lavage, 21 · 0 % (15 · 0–27 · 0), compared with expectorated, 7 · 7 % (4 · 4–11 · 1), or induced sputum, 11 · 7 % (4 · 9–18 · 4). Polymerase chain reaction (PCR) was used in 14 studies (*n* = 1686). There was a trend of decreasing PCP prevalence amongst inpatients over time, from 28 % (21–34) in the 1990s to 9 % (8–10) after 2005. The case fatality rate was 18 · 8 % (11 · 0–26 · 5), and PCP accounted for 6 · 5 % (3 · 7–9 · 3) of study deaths.

**Conclusions:**

PCP is an important opportunistic infection amongst HIV-infected adults in sub-Saharan Africa, particularly amongst patients admitted to hospital. Although prevalence appears to be decreasing, improved access to antiretroviral therapy and non-invasive diagnostics, such as PCR, are needed.

**Electronic supplementary material:**

The online version of this article (doi:10.1186/s12879-016-1809-3) contains supplementary material, which is available to authorized users.

## Background

*Pneumocystis jirovecii* is a ubiquitous opportunistic fungal pathogen that causes pneumocystis pneumonia (PCP) in patients with cellular immune defects. PCP heralded the onset of the global human immunodeficiency virus (HIV) pandemic [1], and prior to 1995 it was estimated that two-thirds of HIV-infected persons would eventually develop PCP [2]. Although the incidence has declined since the introduction of cotrimoxazole prophylaxis and combination antiretroviral therapy (ART), PCP remains the most important AIDS-defining opportunistic infection in the United States [3, 4]. By contrast, in sub-Saharan Africa the morbidity and mortality in HIV is dominated by tuberculosis [5], cryptococcal disease, and bacterial infections such as non-typhoidal salmonella and *Streptococcus pneumoniae* [6].

Early studies from African countries with high HIV prevalence reported PCP to be an uncommon OI [7–9]. An overwhelmingly high burden of other opportunistic diseases, early exposure to *P. jirovecii* resulting in improved immunity, and immune modulation by other infections are putative explanations for the reduced prevalence of PCP in sub-Saharan Africa [10–12]. A single recent study demonstrated an inverse relationship of PCP prevalence to gross domestic product [13].

In the context of late presentation at low CD4 T lymphocyte counts (CD4 count) [14] and limited ART coverage [15], there is potentially a large pool of individuals at high risk of presenting to health care facilities with PCP in sub-Saharan Africa. Local studies have revealed a high seroprevalence of *P. jirovecii* in healthy HIV-infected adults [16, 17], as well as high rates of clinical disease in African children [18–20]. The reported increase in prevalence amongst adults in the 1990s, possibly as a result of better access to diagnostics and treatment [21], may reflect the true epidemiology of the disease, suggesting that PCP may be a more important cause of community-acquired pneumonia in Africa than was previously recognised.

A better understanding of PCP prevalence will help to inform empiric prescribing, the interpretation of diagnostic tests, and public health policy relating to the allocation of scarce resources such as intensive care unit beds, invasive procedures like bronchoscopy, and research funding. We therefore undertook a systematic review to more accurately determine the burden of PCP in sub-Saharan Africa in the ART era. Our primary aim was to determine the proportion of HIV-infected adults with confirmed PCP, stratified by clinical setting, method of diagnosis, and time. Secondary aims included attributable mortality and case fatality, a description of demographic profiles, and rates of co-infection with other pathogens.

## Methods

### Search strategy and selection criteria

A detailed protocol describing our methods has been published elsewhere [22] and is registered in the Prospero database (CRD42013005530). We developed a broad search strategy to identify studies that reported the prevalence, mortality or case fatality of PCP in sub-Saharan Africa. Our systematic review used the search terms “pneumocystis,” “*P jirovecii*,” “*P carinii*,” “pneumonia,” “HIV,” “Africa south of the Sahara,” and related terms for relevant studies published in Pubmed, Web of Science, Africa-Wide: NiPAD and CINAHL between 1995 and 1 June 2015 (the full search strategy is included in the supplementary appendix, Additional file 1). No language or age limits were applied. We hand-searched bibliographies of all recovered articles for potentially eligible studies and contacted authors for unpublished data. The year 1995 was chosen as a cut-off because previous reviews have reported on PCP prevalence from early studies in sub-Saharan Africa, and we wanted to assess the impact of more widespread availability of cotrimoxazole prophylaxis and ART. Titles and abstracts of references recovered by the search were screened and the full texts of potentially relevant articles were independently assessed by two reviewers (SW and RG) using a standardised score sheet. Disagreements on final inclusions were resolved by discussion and consensus involving a third reviewer (MM).

Studies assessing a clearly defined population of HIV-infected adults (≥13 years of age, chosen because the diagnosis and treatment of PCP in this age group is identical to older adults, and 13 years is the age of transition into adult care in the public health sector in South Africa) in any clinical setting were included if they applied specific diagnostic criteria for PCP. These were predefined with the use of a novel quality-scoring tool, and included either a clear clinical case definition (based on World Health Organization criteria [23]) or confirmation with laboratory testing (using histochemical or fluorescent staining or molecular assays). Reports involving cohorts of patients with PCP were ineligible for primary analysis of pooled prevalence, but were included if they provided data on case fatality.

### Data extraction

Data were independently extracted by two reviewers (SW and RG) and compared for consistency using pre-specified scores derived for each main eligibility category. The key variable was the proportion of HIV-infected adults in the study cohort diagnosed with PCP. As a minority of studies were specifically designed to assess this outcome, our denominator was defined as the population of patients who underwent clinical or laboratory evaluation for PCP. Prevalence was defined as the number of cases of PCP diagnosed among patients with signs and symptoms of PCP who would normally warrant testing at a given hospital or clinic, and not the population prevalence of disease. We defined mortality and case fatality as the proportion of deaths due to PCP (PCP deaths/total deaths) and mortality amongst PCP cases (PCP deaths/total PCP diagnoses), respectively.

Data were stratified by clinical setting. As an indicator of severity and as a crude estimate of the pre-test probability of PCP, we categorised in- and outpatients into separate groups, given that susceptible inpatients with respiratory symptoms are more likely to have a diagnosis of PCP and to have more severe disease than outpatients, which will influence PCP prevalence and mortality. Studies that recruited both in- and outpatients, but from which the respective proportions of these groups could not be ascertained, were classified separately. We also stratified data by diagnostic method and clinical specimen.

Patients with symptoms compatible with PCP plus any positive diagnostic test or fulfilling criteria for the clinical case definition were considered to have confirmed disease, and were included in our primary analysis. Asymptomatic individuals or those with an illness that resolved without specific PCP therapy, but had a positive *P. jirovecii* PCR (any assay) were defined as being colonised.

Where available, the following information was captured; CD4 count, numbers of patients on cotrimoxazole prophylaxis and/or ART, type and frequency of co-infection(s), relevant data from studies with duplicate cohorts that were excluded from the pooled prevalence analysis, and demographic details.

### Methodological quality of included studies

Overall quality of included studies was evaluated using our published scoring system [22]. The tool assigns numerical scores to the following domains: participant selection (study design and objectives), outcome ascertainment (diagnostic criteria) and quality of data available for extraction. We used the tool to code and standardise study eligibility decisions and to assess agreement between investigators. As this is a prevalence review, we assessed studies for selection bias and overall bias, using criteria defined by the Cochrane group (Review Manager Version 5.2, http://ims.cochrane.org/RevMan).

### Data analysis

Point estimates and 95 % confidence intervals were derived for all outcomes using prevalence data from included studies. Meta-analysis was performed according to the Mantel-Haenszel method applying the random-effects model to account for between study variability. We evaluated heterogeneity using the *χ*2-based Q statistic (significant for *p* <0 · 1) and the I^2^ statistic (>50 % indicative of “notable” heterogeneity) [24]. STATA software version 11 (STATA Corporation, College Station, Texas) was used to perform calculations and the meta-analysis and to produce the forest plots using the *metan* routine. Where standard errors (SE) were not provided, we incorporated confidence intervals into the formula, SE = (upper limit – lower limit)/3 · 92.

## Results

### Study characteristics

**Fig. 1:**
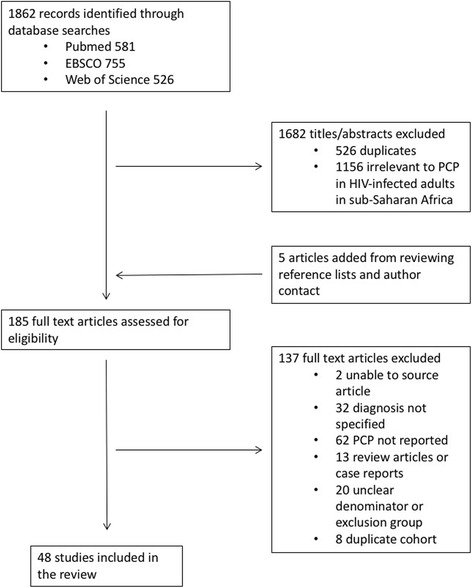
PRISMA flow diagram

Our search identified 1862 records, with an additional five studies added from searching bibliographies. After removing duplicates and screening abstracts for relevance to the study aims, we evaluated 185 full text articles for eligibility, ultimately including 48 unique study populations (Fig. 1; a full reference list of included studies available in the online supplementary appendix, Additional file 2). Of these, 35 studies (6018 patients) reported the prevalence of PCP from clinical cohorts; eleven were post-mortem studies (707 deaths) and two provided data on clinical characteristics and case fatality only. For the studies with a clear denominator to evaluate PCP prevalence, the pre-defined clinical categories were represented as follows: inpatients, 23 studies (*n* = 2593); outpatients, 7 studies (*n* = 2656); and enrolment of both in- and outpatients, 5 studies (*n* = 769). The dataset included patients from 18 sub-Saharan African countries: Benin, Burkina Faso, Central African Republic, Mali, Mozambiqe, Nigeria, Namibia and Zambia (1 study); Botswana and Zimbabwe (2 studies); Côte d’ Iviore, Ethiopia, Malawi, Senegal and Tanzania (3 studies); Kenya (4 studies); Uganda (8 studies); South Africa (13 studies). Apart from 3 clinical studies that recruited patients from district hospitals, all others were conducted in tertiary or central hospital referral centres.

Study characteristics are summarised in the online supplementary appendix (Additional file 3). The median CD4 count of patients assessed for PCP was 75 cells/mm^3^ (interquartile range﻿ (IQR) 63–145; 20 studies, *n* = 3543). 18 · 7 % (983/5249) were on ART (26 studies, *n* = 5249) and 34 · 4 % (1377/4002) were receiving PCP prophylaxis (21 studies, *n* = 4002).

Although most studies (42, *n* = 6285) were prospective in design the overall data quality was poor: risk of bias was assessed as high in 30 studies (*n* = 4346) and 19 studies (*n* = 4140) were assigned an overall quality assessment score of ≤ 9 (poor quality) (available in the online supplementary appendix, Additional file 4). Agreement between investigators with regard to decisions about applied qualitative scores was acceptable (interrater agreement = 95 · 12 %, kappa = 0 · 8292).

### PCP prevalence

**Fig. 2:**
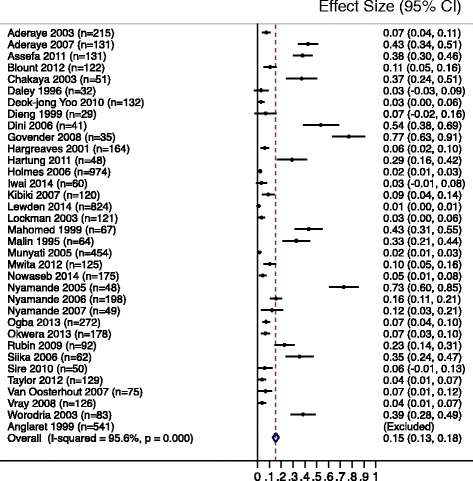
Pooled prevalence of PCP across all clinical settings

The overall prevalence of PCP among 6018 patients in 35 clinical studies was 15 · 4 % (95 % confidence interval (CI), 12 · 9–18 · 0) (Fig. 2). Heterogeneity was high (*p* < 0 · 1; I^2^ = 95 · 6 %). Among all patients presenting with respiratory symptoms the prevalence was 18 · 8 % (31 studies, *n* = 3504, 95 % CI, 14 · 9–22 · 6), and 17 · 1 % (21 studies, *n* = 2086, 95 % CI 12 · 5–21 · 7) in studies that evaluated patients who were sputum smear-negative for acid-fast bacilli.

**Fig. 3:**
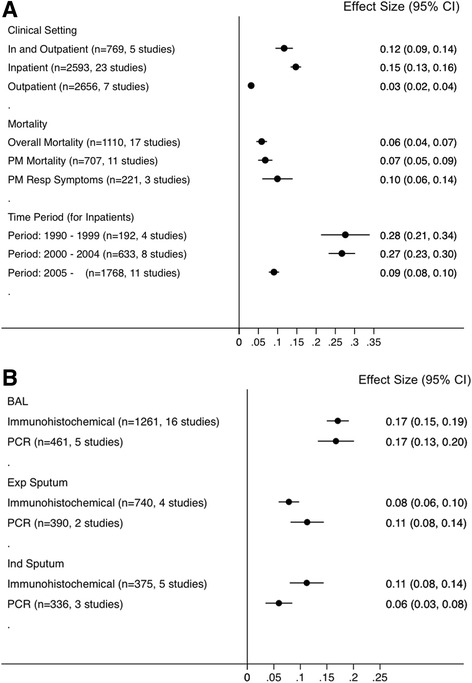
Forest plots of proportion of patients with PCP by clinical setting, mortality and time period (**a**) and by diagnostic method (**b**). *PM* post mortem, *resp* respiratory *BAL* bronchoalveolar lavage, *PCR* polymerase chain reaction, *Exp* expectorated, *Ind* induced. Heterogeneity: A: Clinical Setting, *p* < 0.1; I^2^ = 99.4 %; Mortality, *p* = 0.83; I^2^ = 0.0 %; Time Period, *p* < 0.1; I^2^ = 91.8 %. B: BAL, *p* = 0.62; I^2^ = 0.0 %; expectorated sputum, *p* = 0.51; I^2^ = 0.0 %; induced sputum, *p* < 0.1; I^2^ = 71.9 %

The proportions of PCP cases stratified by clinical setting, post mortem reports, time period studied and diagnostic methods used, are shown in Fig. 3. By setting, prevalence was highest amongst inpatients (22 · 4 %, 23 studies, *n* = 2593, 95 % CI 17 · 2–27 · 7) compared with outpatients (4 · 8 %, 7 studies, *n* = 2656, 95 % CI 2 · 7–6 · 9) and patients recruited from both in- and outpatient settings (11 · 5 %, *n* = 769, 95 % CI 4 · 1–19 · 0). PCP was diagnosed in 24 % (22 studies, *n* = 1769, 95 % CI 17 · 8–30 · 3) of inpatients with respiratory symptoms.

Figure 3a shows the comparison of PCP prevalence for inpatients by the year that data was last collected in the study. The pooled percentages of PCP cases amongst inpatients over three time periods were as follows: 1990s, 28 % (4 studies, *n* = 192, 95 % CI 21–34), 2000–2004, 27 % (8 studies, *n* = 633, 95 % CI 23–30), 2005 and later, 9 % (11 studies, *n* = 1768, 95 % CI 8–10).

The proportion of inpatients on either ART or cotrimoxazole in the two earlier time periods was 3 · 6 % (*n* = 276, 95 % CI 1 · 8–6 · 6) and 2 · 6 % (*n* = 227, 95 % CI 0 · 98–5 · 7) respectively, and the proportions in the years after 2005 were 24 · 3 % (*n* = 1482, 95 % CI 9 · 8–38 · 7) and 30 · 4 % (*n* = 1421, 95 % CI 11 · 6–49 · 3) respectively.

PCP prevalence according to diagnostic method is shown in Fig. 3b. The prevalence for clinical specimens diagnosed by BAL was 21 · 0 % (*n* = 1591, 95 % CI 15 · 0–27 · 0); this was significantly higher than from expectorated sputum (7 · 7 %, *n* = 740, 95 % CI 4 · 4–11 · 1), and numerically higher than from induced sputum (11 · 7 %, *n* =553, 95 % CI 4 · 9–18 · 4). The type of clinical specimen was not specified in 3 studies (*n* = 445, PCP cases 40).

### Case fatality, mortality and clinical characteristics

The clinical characteristics of PCP cases, proportions and type of co-infections are listed in table 1. The case fatality was 18 · 8 % (95 % CI 11 · 0–26 · 5; range 0–71 · 4 %); data for this calculation were available from 12 studies (*n* = 334), representing fewer than half (334/716) of all confirmed cases. Only two studies (*n* = 16) reported follow up times; 11 patients were followed for 1 month (0 deaths) and five followed for 2 months post-discharge (case fatality 60 %). All other PCP-related deaths occurred during the index admission. 29 · 5 % of the 474 PCP cases in whom additional testing was performed had a confirmed co-existent opportunistic pulmonary disease. Tuberculosis was most common (14 · 8 %), followed by bacterial pneumonia (8 · 7 %) and Kaposi sarcoma (4 · 1 %).

**Table 1:** Characteristics of all PCP cases (*n* = 743)

Age (years, mean)	35 (10 studies, *n* = 240)
Male sex (%)	39.6 (15 studies, *n* = 315)
CD4 count (cells/microL, median, IQR)	48 [31.5–129.0] (16 studies, *n* = 353)
Cotrimoxazole prophylaxis (%, 95 % CI)	5.9 [3.3–9.5] (11 studies, *n* = 255)
ART (%, 95 % CI)	0.4 [0.01–2.6] (11 studies, *n* = 215)
Co-existent opportunistic disease (%, 95 % CI)	
- Overall	29.3 [25.4–33.6] (26 studies, *n* = 474)
- Tuberculosis	14.8 [11.8–18.5] (25 studies, *n* = 431)
- Bacterial pneumonia	8.7 [0.6–11.8] (22 studies, *n* = 445)
- Pulmonary cryptococcosis	1.4 [0.4–3.6] (17 studies, *n* = 283)
- Pulmonary Kaposi sarcoma	4.1 [2.4–6.6] (21 studies, *n* = 410)
- Pulmonary CMV	3.9 [1.4–8.2] (11 studies, *n* = 155)
- Pulmonary MAC	0.8 [0.1–2.8] (19 studies, *n* = 254)
Case fatality rate (%, 95 % CI)	18.8 % [11.0–26.5] (12 studies, *n* = 334)

*IQR* interquartile range; *CI* confidence interval; *ART* antiretroviral therapy; *CMV* cytomegalovirus; *MAC* mycobacterium avium complex

Where data were available, PCP accounted for 6 · 5 % (95 % CI 3 · 7–9 · 3) of the overall mortality (17 studies, *n* = 1110). Post-mortem studies provided data for 707 deaths of which 5 · 9 % (95 % CI 3 · 2–8 · 6) were attributed to PCP. Apart from one study that evaluated all-cause maternal mortality, the primary objectives of other post-mortem studies included the determination of cause of death in HIV-infected adults. Three of these studies (*n* = 221) enrolled subjects with pre-morbid respiratory symptoms, in whom PCP-related mortality was 10 % (95 % CI 6–14).

## Discussion

Early studies conducted in sub-Saharan Africa suggested a lower PCP prevalence than in developed countries [25–27], as well as a lower incidence (5 cases per 1000 person years) compared to the post-ART era estimate in the United States of 46 cases per 1000 person years [28]. These data have led a number of experts to question the importance of PCP in comparison to tuberculosis and other infections that dominate mortality and morbidity in sub-Saharan Africa [10–12]. Our review of data collected predominantly after 1995 demonstrates a pooled prevalence of PCP comparable to that reported from developed countries [29], suggesting that it is more likely to have been missed or neglected in sub-Saharan Africa rather than being an uncommon opportunistic infection.

Previous reviews have shown time trend of increasing PCP prevalence in sub-Saharan Africa amongst comparable patient populations and diagnostic assessments. After crudely combining the data from these reports, seven out of ten studies conducted over the period 1986–1993 had prevalence rates ≤ 15 % while eight out of ten studies conducted between 1997 and 2005 had prevalence rates ≥ 30 % [21, 30]. We have shown the opposite, that the proportion of inpatients with PCP appears to be decreasing over time. This trend seems more realistic, and it is plausible that our observation of better exposure to ART and cotrimoxazole prophylaxis in more recent years can account for fewer diagnosed cases of PCP. Nonetheless, even after 2005, most patients in our included studies had not received these interventions and had low CD4 counts at presentation, reflecting the slow rollout of adequate HIV care in the region.

We noted a much higher prevalence of PCP amongst inpatients compared to outpatients. This is mainly a reflection of the superior performance of invasive diagnostic tests and that inpatients represent a selected denominator of patients that have failed empiric therapy for other causes of pneumonia in the community. However, the observation may also suggest something inherent about PCP itself, possibly that mild cases either resolve spontaneously or are an uncommon manifestation of the infection; these hypotheses require further study.

More cases of PCP were diagnosed using BAL (21 %) compared with sputum (7–11 %). This reflects a selection bias of patients with a higher likelihood of PCP being referred for bronchoscopy but also the enhanced sensitivity of BAL for diagnosis. Unfortunately, access to bronchoscopy is extremely limited in most African countries, and clinicians must rely on a combination of knowledge of disease prevalence, unvalidated clinical case definitions and insensitive non-invasive tests to diagnose PCP. In the context of a prevalence of > 20 % and poor diagnostic tools, there should be a low threshold to initiate empiric PCP therapy for HIV-infected inpatients with pneumonia. This may lead to overtreatment, with the attendant risks of unnecessary exposure to high dose cotrimoxazole and concerns about using corticosteroids in patients with undiagnosed tuberculosis or Kaposi sarcoma. Our finding of a high frequency of PCP with the use of bronchoscopy therefore emphasises the need for more accurate non-invasive diagnostic tests such as PCR, which has been shown to be sensitive, specific and cost effective when used on sputum and BAL [31], with a potentially important role as a rule-out test for PCP in resource-limited settings [32]. Only ten studies included in our review used PCR assays to diagnose PCP, and similar numbers of cases were identified by this method and staining techniques on all specimen types. Our study was not designed to assess the performance of diagnostic tests for PCP, and prospective cohort studies are needed to evaluate the role of PCR, and other non-invasive tests such as plasma beta-glucan [33], in the diagnosis of PCP in sub-Saharan Africa.

Our calculated case fatality of almost 19 % is higher than the mortality from other common respiratory infections in Africa [34, 35] as well as from PCP in developed countries [36]. This contributes to the overall burden of PCP and adds impetus to more investment in prevention strategies and effective diagnostic tests.

Similarly to a recent review [13], a substantial number of patients with PCP were found to have coexistent bacterial pneumonia and other opportunistic infections, particularly tuberculosis. This is an important consideration when evaluating patients with inadequate responses to empiric or confirmed PCP therapy, and co-infections should be excluded before attributing a poor treatment response to PCP infection alone.

This review has a number of important limitations. First, most included studies were conducted in highly selected cohorts, such as patients with suspected PCP, exclusion of patients with tuberculosis and other illnesses, and the performance of BAL only in cases where the diagnosis remained elusive. This increases the risk of selection bias and of overestimating true disease prevalence. However, the clinical burden of PCP in a population is influenced almost exclusively by those who are ill with respiratory symptoms and our chosen denominator therefore allows an assessment that is probably close to the true prevalence.

Second, missing or unreported data was common. With regard to disease prevalence, bronchoscopy studies frequently excluded a substantial proportion of patients with possible PCP on the basis of medical contraindications for the procedure, and five studies excluded patients with either advanced HIV or severe respiratory illness. If anything, this may result in an underestimate of PCP prevalence because the outcomes of otherwise eligible patients with respiratory symptoms not enrolled were rarely reported and over half of the included studies did not specifically evaluate participants for PCP.

The third limitation was the high degree of heterogeneity in the prevalence findings from different studies. This is not unusual in prevalence reviews, and reflects differences in methodology and aims, diagnostic methods and the chosen population denominator. We attempted to minimise this effect by analysing data by clinical setting to include patients with similar risk for PCP and by scoring the quality of each study, and found no significant difference in prevalence on the basis of study quality (data not shown). It is also possible that some undefined environmental or biological differences exist across geographic locations to explain the observed heterogeneity, but our study was not designed to address this.

## Conclusions

We have performed a systematic review of PCP prevalence using diagnostic criteria with high specificity, a well-defined population denominator and clinically relevant categorisation of cases. The overall impression from our pooled data is that PCP is an important cause of community-acquired pneumonia in HIV-infected adults living in sub-Saharan Africa. This has implications for clinical guidelines in resource poor settings where indications for empiric therapy and referral need to be clearly defined. The contribution of PCP to mortality in the region suggests that cases are unrecognised, and highlights the need for the development of a reliable clinical case definition and the evaluation of sensitive non-invasive diagnostic tests, such as PCR. The observed trend of decreasing PCP prevalence is encouraging, but there is much work to be done to expand access to ART and cotrimoxazole prophylaxis so that ultimately this sentinel AIDS-defining infection achieves the status of a rare disease it was once erroneously ascribed.

## Additional files

Additional file 1: Table S1. Full search strategy. (PDF 817 kb) Additional file 2: Full reference list of included studies. (DOCX 121 kb) Additional file 3: Summary table of included study cohorts. (DOCX 158 kb) Additional file 4: Figure showing bias and quality assessment. (PPTX 93 kb)
