# Supplementary material for: Burden of pneumocystis pneumonia in HIV-infected adults in sub-Saharan Africa: a systematic review and meta-analysis
Source: BMC Infect Dis. 2016 Sep 9;16(1):482. doi: 10.1186/s12879-016-1809-3 (PMC5018169; doi:10.1186/s12879-016-1809-3)
Supplement: Additional file 1: Table S1. — Full search strategy. (PDF 817 kb) [file 12879_2016_1809_MOESM1_ESM.pdf]

Table S1: SEARCH STRATEGY

SEARCH	MeSH term (modified as needed for use in other databases)
#1	pneumocystis
#2	pneumonia, pneumocystis
#3	pneumocystis jirovecii
#4	pneumocystis carinii
#5	#1 OR #2 OR #3 OR #4
#6	pneumonia
#7	community-acquired pneumonia
#8	bronchopneumonia
#9	#6 OR #7 OR #8
#10	#5 OR #9
#11	hiv
#12	hiv infections
#13	hiv-1
#14	hiv seropositivity
#15	aids-related opportunistic infections
#16	#11 OR #12 OR #13 OR #14 OR #15
#17	acquired immunodeficiency syndrome
#18	aids serodiagnosis
#19	#17 OR #18
#20	#16 OR #19
#21	africa
#22	africa south of the sahara
#23	africa, western
#24	africa, southern
#25	africa, eastern
#26	africa, central
#27	#21 OR #22 OR #23 OR #24 OR #25 OR #26
#28	angola OR benin OR botswana OR burkina faso OR burundi OR central african republic OR chad OR congo OR cote d ivoire OR democratic republic of the congo OR djibouti OR ethiopia OR eritrea OR equatorial guinea OR gabon OR gambia OR ghana OR guinea OR guinea-bissau OR kenya OR lesotho OR liberia OR malawi OR mali OR Mauritania OR mozambique OR namibia OR niger OR nigeria OR rwanda OR senegal OR sierra leone OR somalia OR south africa OR sudan OR swaziland OR tanzania OR togo OR uganda OR united republic of cameroon OR zaire OR zambia OR zimbabwe
#29	#27 OR #28
#30	#10 AND #20 AND #29
