# Supplementary material for: Burden of pneumocystis pneumonia in HIV-infected adults in sub-Saharan Africa: a systematic review and meta-analysis
Source: BMC Infect Dis. 2016 Sep 9;16(1):482. doi: 10.1186/s12879-016-1809-3 (PMC5018169; doi:10.1186/s12879-016-1809-3)
Supplement: Additional file 2: — Full reference list of included studies. (DOCX 121 kb) [file 12879_2016_1809_MOESM2_ESM.docx]

**Citations for included studies**

1. Aderaye G, Bruchfeld J, Aseffa G, et al. Pneumocystis jiroveci pneumonia and other pulmonary infections in TB smear-negative HIV-positive patients with atypical chest X-ray in Ethiopia. *Scand J Infect Dis* 2007; **39**(11-12): 1045-53.

2. Aderaye G, Bruchfeld J, Olsson M, Lindquist L. Occurrence of Pneumocystis carinii in HIV-positive patients with suspected pulmonary tuberculosis in Ethiopia. *Aids* 2003; **17**(3): 435-40.

3. Anglaret X, Chêne G, Attia A, et al. Early chemoprophylaxis with trimethoprim-sulphamethoxazole for HIV-1-infected adults in Abidjan, Côte d'Ivoire: a randomised trial. *The Lancet* 1999; **353**(9163): 1463-8.

4. Ansari NA, Kombe AH, Kenyon TA, et al. Pathology and causes of death in a group of 128 predominantly HIV-positive patients in Botswana, 1997-1998. *Int J Tuberc Lung Dis* 2002; **6**(1): 55-63.

5. Assefa GN, Y.; Aderaye,G.; Worku,A.; Lindquist,L. Chest X-ray evaluation of pneumonia-like syndromes in smear negative HIV-positive patients with atypical chest x-ray. Findings in Ethiopian setting. *Ethiop Med J* 2011; **49**(1): 35 - 42.

6. Bates M, Mudenda V, Shibemba A, et al. Burden of tuberculosis at post mortem in inpatients at a tertiary referral centre in sub-Saharan Africa: a prospective descriptive autopsy study. *Lancet Infectious Diseases* 2015; **15**(5): 544-51.

7. Blount RJ, Jarlsberg LG, Daly KR, et al. Serologic responses to recombinant Pneumocystis jirovecii major surface glycoprotein among Ugandan patients with respiratory symptoms. *PloS one* 2012; **7**(12): e51545.

8. Chakaya JM, Bii C, Ng'ang'a L, et al. Pneumocystis carinii pneumonia in HIV/AIDS patients at an urban district hospital in Kenya. *East Afr Med J,* 2003; **80**(1): 30 - 5.

9. Cox JA, Lukande RL, Kalungi S, et al. Needle Autopsy to Establish the Cause of Death in HIV-Infected Hospitalized Adults in Uganda: A Comparison to Complete Autopsy. *Jaids-Journal of Acquired Immune Deficiency Syndromes* 2014; **67**(2): 169-76.

10. Cox JA, Lukande RL, Nelson AM, et al. An autopsy study describing causes of death and comparing clinico-pathological findings among hospitalized patients in Kampala, Uganda. *PloS one* 2012; **7**(3): e33685.

11. Daley CL, Mugusi F, Chen LL, et al. Pulmonary complications of HIV infection in Dar es Salaam, Tanzania. Role of bronchoscopy and bronchoalveolar lavage. *American journal of respiratory and critical care medicine* 1996; **154**(1): 105-10.

12. Deok-jong Yoo S, Worodria W, Davis JL, et al. The prevalence and clinical course of HIV-associated pulmonary cryptococcosis in Uganda. *Journal of acquired immune deficiency syndromes* 2010; **54**(3): 269-74.

13. Dieng Y, Ndour A, Gaye O, et al. Pneumocystosis in HIV infected patients presenting with acid-fast bacilli negative pneumopathy at the Central University Hospital at Dakar. *Dakar Med* 1999; **44**(1): 28 - 31.

14. Dini L, Du Plessis M, Wong M, Karstaedt A, Fernandez V, Frean J. Prevalence of DHPS polymorphisms associated with sulfa resistance in South African Pneumocystis jirovecii strains. *The Journal of eukaryotic microbiology* 2006; **53 Suppl 1**: S110-1.

15. Domoua K, N'Dhatz M, Coulibaly G, et al. Autopsy findings in 70 AIDS patients who died in a department of pneumology in Ivory Coast: impact of tuberculosis. *Med Trop* 1995; **55**(3): 252 - 4.

16. Govender S, du Plessis SJ, Ocana GS, Chalkley LJ. Prevalence of Pneumocystis jirovecii and Mycoplasma pneumoniae in patients presenting with pneumonia at hospitals in Port Elizabeth. *Southern African Journal of Epidemiology and Infection,* 2008; **23**(2): 21 - 4.

17. Hargreaves NJ, Kadzakumanja O, Phiri S, et al. Pneumocystis carinii pneumonia in patients being registered for smear-negative pulmonary tuberculosis in Malawi. *Transactions of the Royal Society of Tropical Medicine and Hygiene* 2001; **95**(4): 402-8.

18. Hartung TK, Chimbayo D, van Oosterhout JJ, et al. Etiology of suspected pneumonia in adults admitted to a high-dependency unit in Blantyre, Malawi. *The American journal of tropical medicine and hygiene* 2011; **85**(1): 105-12.

19. Holmes CB, Wood R, Badri M, et al. CD4 decline and incidence of opportunistic infections in Cape Town, South Africa: implications for prophylaxis and treatment. *Journal of acquired immune deficiency syndromes* 2006; **42**(4): 464-9.

20. Iwai S, Huang D, Fong S, et al. The Lung Microbiome of Ugandan HIV-Infected Pneumonia Patients Is Compositionally and Functionally Distinct from That of San Franciscan Patients. *PloS one* 2014; **9**(4): 9.

21. Karstaedt AS, Grannum S. Pneumocystis carinii pneumonia in patients with AIDS in South Africa. *Transactions of the Royal Society of Tropical Medicine and Hygiene* 2001; **95**(1): 40-1.

22. Kibiki GS, Beckers P, Mulder B, et al. Aetiology and presentation of HIV/AIDS-associated pulmonary infections in patients presenting for bronchoscopy at a referral hospital in northern Tanzania. *East African medical journal* 2007; **84**(9): 420 - 8.

23. Lewden C, Drabo YJ, Zannou DM, et al. Disease patterns and causes of death of hospitalized HIV-positive adults in West Africa: a multicountry survey in the antiretroviral treatment era. *Journal of the International Aids Society* 2014; **17**: 12.

24. Lockman S, Hone N, Kenyon TA, et al. Etiology of pulmonary infections in predominantly HIV-infected adults with suspected tuberculosis, Botswana. *Int J Tuberc Lung Dis* 2003; **7**(8): 714-23.

25. Mahomed AG, Murray J, Klempman S, et al. Pneumocystis carinii pneumonia in HIV infected patients from South Africa. *East African medical journal* 1999; **76**(2): 80 - 4.

26. Malin AS, Gwanzura LK, Klein S, Robertson VJ, Musvaire P, Mason PR. Pneumocystis carinii pneumonia in Zimbabwe. *Lancet* 1995; **346**(8985): 1258-61.

27. Martinson NA, Karstaedt A, Venter WD, et al. Causes of death in hospitalized adults with a premortem diagnosis of tuberculosis: an autopsy study. *Aids* 2007; **21**(15): 2043-50.

28. Menendez C, Romagosa C, Ismail MR, et al. An autopsy study of maternal mortality in Mozambique: the contribution of infectious diseases. *PLoS Med* 2008; **5**(2): e44.

29. Munyati SS, Dhoba T, Makanza ED, et al. Chronic cough in primary health care attendees, Harare, Zimbabwe: diagnosis and impact of HIV infection. *Clinical infectious diseases : an official publication of the Infectious Diseases Society of America* 2005; **40**(12): 1818-27.

30. Murray J, Sonnenberg P, Nelson G, Bester A, Shearer S, Glynn JR. Cause of death and presence of respiratory disease at autopsy in an HIV-1 seroconversion cohort of southern African gold miners. *Aids* 2007; **21 Suppl 6**: S97-S104.

31. Mwita J, Mugusi F, Pallangyo K. Pneumocyctis pneumonia and pulmonary tuberculosis among HIV-infected patients at Muhimbili National Hospital, Tanzania. *East Afr J Public Health* 2012; **9**(1): 10-2.

32. Nowaseb V, Gaeb E, Fraczek MG, Richardson MD, Denning DW. Frequency of Pneumocystis jirovecii in sputum from HIV and TB patients in Namibia. *Journal of Infection in Developing Countries* 2014; **8**(3): 349-57.

33. Nyamande K, Lalloo UG. Serum procalcitonin distinguishes CAP due to bacteria, Mycobacterium tuberculosis and PJP. *Int J Tuberc Lung Dis* 2006; **10**(5): 510-5.

34. Nyamande K, Lalloo UG, Vawda F. Comparison of plain chest radiography and high-resolution CT in human immunodeficiency virus infected patients with community-acquired pneumonia: a sub-Saharan Africa study. *Br J Radiol* 2007; **80**(953): 302-6.

35. Nyamande K, Lalloo UG, York D, Naidoo M, Irusen EM, Chetty R. Low sensitivity of a nested polymerase chain reaction in oropharyngeal washings for the diagnosis of pneumocystis pneumonia in HIV-infected patients. *Chest* 2005; **128**(1): 167-71.

36. Ogba OM, Abia-Bassey LN, Epoke J. The relationship between opportunistic pulmonary fungal infections and CD4 count levels among HIV-seropositive patients in Calabar, Nigeria. *Transactions of the Royal Society of Tropical Medicine and Hygiene* 2013; **107**(3): 170-5.

37. Okwera A, Bwanga F, Najjingo I, et al. Aetiology of pulmonary symptoms in HIV-infected smear negative recurrent PTB suspects in Kampala, Uganda: a cross-sectional study. *PloS one* 2013; **8**(12): e82257.

38. Orlovic D, Kularatne R, Ferraz V, Smego RA, Jr. Dual pulmonary infection with Mycobacterium tuberculosis and Pneumocystis carinii in patients infected with human immunodeficiency virus. *Clinical infectious diseases* 2001; **32**(2): 289-94.

39. Rana F, Hawken MP, Meme HK, et al. Autopsy findings in HIV-1-infected adults in Kenya. *J Acquir Immune Defic Syndr Hum Retrovirol* 1997; **14**(1): 83-5.

40. Rana FS, Hawken MP, Mwachari C, et al. Autopsy study of HIV-1-positive and HIV-1-negative adult medical patients in Nairobi, Kenya. *Journal of acquired immune deficiency syndromes* 2000; **24**(1): 23-9.

41. Rubin G. The spectrum of radiological appearances in brochoscopically proven pneumocystis pneumonia in HIV positive adults: A retrospective analysis from Helen Joseph Hospital. Johannesburg: University of the Witwatersrand; 2011.

42. Siika AM, Chakaya JM, Revathi G, Mohamed SS, Bhatt KM. Bronchoscopic study on aetiology of chronic cough in HIV-infected adults with negative sputum smears for Mycobacterium tuberculosis at Kenyatta National Hospital, Nairobi. *East African medical journal* 2006; **83**(6): 295 - 305.

43. Sire JM, Sow PS, Chartier L. Aetiology of AFB negative pneumonias in hospitalized HIV patients in Dakar. *Rev Mal Respir* 2010; **27**(9): 1015- 21.

44. Taylor SM, Meshnick SR, Worodria W, et al. Low prevalence of Pneumocystis pneumonia (PCP) but high prevalence of pneumocystis dihydropteroate synthase (dhps) gene mutations in HIV-infected persons in Uganda. *PloS one* 2012; **7**(11): e49991.

45. van Oosterhout JJ, Laufer MK, Perez MA, et al. Pneumocystis pneumonia in HIV-positive adults, Malawi. *Emerging infectious diseases* 2007; **13**(2): 325-8.

46. Vray M, Germani Y, Chan S, et al. Clinical features and etiology of pneumonia in acid-fast bacillus sputum smear-negative HIV-infected patients hospitalized in Asia and Africa. *Aids* 2008; **22**(11): 1323-32.

47. Wong EB, Omar T, Setlhako GJ, et al. Causes of death on antiretroviral therapy: a post-mortem study from South Africa. *PloS one* 2012; **7**(10): e47542.

48. Worodria W, Okot-Nwang M, Yoo SD, Aisu T. Causes of lower respiratory infection in HIV-infected Ugandan adults who are sputum AFB smear-negative. *Int J Tuberc Lung Dis* 2003; **7**(2): 117-23.
