# Supplementary material for: Burden of pneumocystis pneumonia in HIV-infected adults in sub-Saharan Africa: a systematic review and meta-analysis
Source: BMC Infect Dis. 2016 Sep 9;16(1):482. doi: 10.1186/s12879-016-1809-3 (PMC5018169; doi:10.1186/s12879-016-1809-3)
Supplement: Additional file 3: — Summary table of included study cohorts. (DOCX 158 kb) [file 12879_2016_1809_MOESM3_ESM.docx]

**Inpatients**

| **Study** | **Period** | **Location** | **Design and objective** | **Setting** | **Participants** | **Diagnosis** | **CD4 count** | **ART** | **PCP prophylaxis** | **Bias** | **Quality score** |
| --- | --- | --- | --- | --- | --- | --- | --- | --- | --- | --- | --- |
| Assefa 2011 | 2004 - 2005  (17 months) | Addis Ababa, Ethiopia | Prospective, consecutive. Accuracy of CXR for the diagnosis of OIs. | Central hospital | HIV+ with respiratory symptoms, sputum AFB -ve, no empiric TB treatment, atypical CXR. 369 screened, 131 enrolled. | BAL and sputum: IFA | NS | NS | 6/131 | Low | 10 |
| Blount 2012 | 2007 - 2008  (12 months) | Kampala, Uganda | Prospective, consecutive. Serological responses to *P. jirovecii*; bronchoscopy on referred patients. | Central hospital | Cough > 2 weeks but < 6 months, sputum AFB -ve, not on TB Rx. 467 HIV+ patients; 122 had bronchoscopy (142 referred). | BAL: modifed Giemsa and PCR | Median 50 | 71/467 | 256/466 | High | 9 |
| Chakaya 2003 | 1999 - 2000  (13 months) | Nairobi, Kenya | Prospective, consecutive. Prevalence and outcomes of PCP. | District hospital | HIV+ with respiratory symptoms, bilateral infiltrates on CXR, sputum AFB -ve. 63 referred, 51 patients underwent bronchoscopy | BAL TBO and IFA | NS | NS | NS | Uncertain | 11 |
| Daley 1996 | 1991 - 1993  (17 months) | Dar es Salaam, Tanzania | Prospective, consecutive. Causes of acute respiratory disease. | Referral hospital | New onset lung disease and abnormal CXR; BAL if sputum AFB -ve and no response to antibiotics. 237 enrolled, 127 HIV+; BAL in 32 | BAL: TBO and Giemsa | NS | NS | NS | High | 10 |
| Deok-jong Yoo 2010 | 2007 - 2008  (11 months) | Kampala, Uganda | Prospective, consecutive. Prevalence, features, outcomes of cryptococcal lung disease on BAL. | Referral centre | HIV+ with cough > 2 weeks but < 6 months, pneumonia, not on TB Rx. BAL if AFB –ve. 218 enrolled, bronchoscopy in 132. | BAL: modified Giemsa stain | Median 67 | 22/132 | NS | High | 9 |
| Dieng 1999 | 1996 - 1997  (9 months) | Dakar, Senegal | Prospective, consecutive. PCP prevalence. | Central hospital | HIV+ with respiratory illness, sputum AFB -ve. 29 enrolled. | BAL: TBO and Giemsa | NS | NS | NS | High | 10 |
| Dini 2006 | NS | Johannesburg, South Africa | Prospective, laboratory study, unclear selection. Prevalence of *P. jirovecii* polymorphisms. | NS | HIV+ with suspected PCP. 41 suspected cases included. | Induced sputum: IFA | NS | None | 1/41 | High | 8 |
| Govender 2008 | 2006  (10 months) | Port Elizabeth, South Africa | Prospective, consecutive. Prevalence of PCP and mycoplasma in CAP. | District hospitals | All patients with CAP. 45 recruited, 35 HIV+ adults | Sputum: PCR | NS | NS | NS | High | 8 |
| Hartung 2011 | 2006  (7 months) | Blantyre, Malawi | Prospective, consecutive. Aetiology of severe CAP. | Central hospital | All patients admitted with severe CAP, sputum AFB -ve. 159 admitted, 51 recruited for BAL: 48 HIV+ (33 excluded because too sick). | BAL: IFA and PCR [Definite: IFA+. Probable: PCR+ with improvement with cotrimoxazole] | Mean 121 | 14/48 | 8/48 | High | 9 |
| Iwai 2014 | 2009 - 2010 (12 months) | Kampala, Uganda | Prospective cohort study, unclear selection. Characterisation of respiratory tract microbiome in HIV+ patients with CAP. | Tertiary hospital | HIV+ with acute pneumonia and sputum AFB -ve. 60 underwent bronchoscopy and included. | BAL: Diff Quik | Median  58 | 9/60 | NS | Uncertain | 9 |
| Kibiki 2007 | NS | Kilimanjaro, Tanzania | Prospective, consecutive. Bronchoscopy study of aetiology of pulmonary infection in HIV. | Central hospital | HIV+ with respiratory symptoms and abnormal CXR. Bronchoscopy for no diagnosis or failure of empiric ABx. Pregnant and severe hypoxia excluded. 120 enrolled. | BAL: Giemsa, GMS, IFA and PCR | Median 65 | NS | NS | High | 10 |
| Lewden 2014 | 2010 (6 months) | Benin, Burkina Faso, Coˆte d’Ivoire, Mali and Senegal | Prospective, consecutive. Profile of hospitalised HIV+ patients. | Tertiary hospitals | All newly hospitalised HIV+ patients. 824 new admissions enrolled. | Clinical case definition (WHO criteria) | Median 75 | 423/824 | 392/824 | Low | 9 |
| Mahomed 1999 | 1985 – 1992  (84 months) | Johannesburg, South Africa | Retrospective record review. Spectrum of pulmonary disease in HIV. | Tertiary hospital | Bronchoscopy performed for HIV+ with respiratory illness, negative sputum smear for AFB and *P. jirovecii* stains. 67 HIV+ reviewed. | TBB/BAL: Grocott and TBO | NS | None | None | High | 10 |
| Malin 1995 | 1992 - 1993  (12 months) | Harare, Zimbabwe | Prospective, consecutive. Causes of diffuse pneumonia unresponsive to antibiotics. | Tertiary hospital | HIV+ with respiratory symptoms, bilateral CXR infiltrates, sputum AFB -ve, no response to penicillin. 64 enrolled. | BAL: MS, TBO, and Diff-Quik | Median 183 (n= 29) | NS | NS | Uncertain | 10 |
| Nyamande 2006 | 2000 - 2001  (14 months) | Durban, South Africa | Prospective, consecutive. Procalcitonin to distinguish causes of CAP. | Tertiary hospital | All patients admitted with CAP, no pre-existing lung disease. 266 total, 198 HIV+. | Induced sputum and BAL: IFA. Bronchoscopy and TBB if no cause/response | NS | NS | NS | Low | 8 |
| Nyamande 2005 | 2000  (7 months) | Durban, South Africa | Prospective, consecutive. Diagnostic performance of oral wash PCR for PCP. | Tertiary hospital | HIV+ with respiratory symptoms and CXR infiltrates suggestive of PCP. 50 recruited, bronchoscopy in 48, TBB in 35. | BAL and oral wash: PCR. TBB: GMS | NS | None | None | High | 9 |
| Nyamande 2007 | 2000 - 2001  (12 months) | Durban, South Africa | Prospective, random selection. Utility of CT chest in CAP. | Tertiary hospital | HIV+ patients admitted with CAP. 49 enrolled | Induced and expectorated sputum: IFA | Mean 184 | None | NS | Uncertain | 8 |
| Rubin 2009 | 1996 – 2003  (84 months) | Johannesburg, South Africa | Retrospective record review. Prevalence of PCP and assessment of CXR accuracy. | Referral centre | HIV+ undergoing bronchoscopy for unclear respiratory illness. 92 included. | BAL: grocott | Median 101 | NS | NS | High | 10 |
| Siika 2006 | 2001 - 2002  (12 months) | Nairobi, Kenya | Prospective, consecutive. Aetiology of chronic cough in HIV and sputum AFB -ve. | Tertiary hospital | HIV+, cough > 3 weeks, abnormal CXR, sputum smear -ve x3 for AFB, no chronic lung disease. 160 recruited, 65 underwent bronchoscopy: 62 analysed, BAL in all, TBB in 41/62. | BAL and TBB: IFA | Median 85 | None | None | High | 10 |
| Sire 2010 | 2003 - 2004  (18 months) | Dakar, Senegal | Prospective, consecutive. Causes of pneumonia in HIV with sputum AFB -ve. | Central hospital | HIV+ with respiratory symptoms and bilateral CXR infiltrates. 196 screened, 68 enrolled, bronchoscopy in 50 (performed if no improvement on empiric Rx, bilat infiltrates, clinician decision) | BAL: IFA | Median 62 | 10/68 | 6/68 | High | 9 |
| Taylor 2012 | 2007 - 2008  (11 months) | Kampala, Uganda | Prospective, consecutive. Prevalence of PCP in HIV with CAP. | Central hospital | HIV+ cough > 2 weeks but < 6 months, clinical pneumonia. BAL if sputum smear AFB -ve. 407 enrolled, 218 assessed and 132 underwent bronchoscopy. 129 analysed (specimens missing). | BAL: Giemsa and PCR (sputum not tested) | Median 74 (n = 128) | 22/129 | 77/129 | Uncertain | 11 |
| Vray 2008 | 2002 - 2005  (39 months) | Senegal and Central African Republic | Prospective, consecutive. Causes of CAP in HIV with sputum AFB -ve. | NS | All HIV+ patients admitted with CAP and sputum AFB -ve; advanced HIV excluded. Senegal 70; CAR 101. BAL performed on 50 and 76 respectively. | Induced sputum and BAL: Grocott and IFA | Median: Senegal 62, CAR 199 | Senegal (10/31); CAR (3/20) | Senegal (6/31); CAR (8/20) | High | 8 |
| Worodria 2003 | 1999 - 2000  (4 months) | Kampala, Uganda | Prospective, consecutive. Aetiology of LRTI in HIV with sputum AFB -ve. | Central hospital | HIV+ cough > 3 weeks, abnormal CXR, sputum AFB -ve, excluded if oxygen saturation < 90%. 150 patients enrolled, 83 underwent bronchoscopy. | BAL: IFA | NS | NS | NS | High | 10 |

**Outpatients**

| **Study** | **Period** | **Location** | **Design and objective** | **Setting** | **Participants** | **Diagnosis** | **CD4 count** | **ART** | **PCP prophylaxis** | **Bias** | **Quality score** |
| --- | --- | --- | --- | --- | --- | --- | --- | --- | --- | --- | --- |
| Aderaye 2003 | 1996  (period NS) | Addis Ababa, Ethiopia | Retrospective analysis of prospective, consecutive cohort. Prevalence of PCP and other pulmonary infections in HIV with smear -ve TB. | NS | All patients with suspected PTB. 119 HIV+ TB culture negative; 96 TB culture positive. | Expectorated sputum: IFA and PCR (single and nested) | NS | None (new HIV) | None | Uncertain | 8 |
| Anglaret 1999 | 1996 - 1998  (22 months) | Abidjan, Cote d' Iviore | Randomised clinical trial. Efficacy of cotrimoxazole prophylaxis in decreasing severe clinical events. | Primary care clinics | HIV+ stage 2 or 3 (stage 1 and 4 excluded). 541 randomised, 270 placebo (only 193 followed up for at least 12 months). | Clinical case definition | Mean 322 and 331 | None | 271/541 | High | 9 |
| Holmes 2006 | 1984 - 2000  (192 months) | Cape Town, South Africa | Cohort study, prospective and retrospective. Rate of CD4 count decline and incidence of OIs. | District and tertiary hospitals | All HIV+ patients attending clinics for ≥ 2 visits. 2086 in cohort, 974 enrolled, 398 lost to follow-up. | Clinical case definition | Median 262 | 125/974 (AZT mono-therapy) | NS | High | 9 |
| Munyati 2005 | NS | Harare, Zimbabwe | Prospective cohort study. Aetiology of chronic cough in primary care and impact of HIV. | Primary care clinics | Chronic cough, ambulatory, no TB treatment, no danger signs. 544 enrolled, 454 HIV+ | Induced sputum: IFA and Grocott. Clinical case definition | NS | NS | NS | High | 10 |
| Mwita 2012 | 2006  (6 months) | Dar es Salaam, Tanzania | Prospective, unspecified enrolment. Prevalence of PCP and TB in HIV+ patients with cough. | Tertiary hospital ARV clinics | HIV+ and cough without underlying chronic disease. 125 enrolled. | Induced sputum: TBO and PCR | Mean 186 | 48/125 | 54/125 | Uncertain | 10 |
| Ogba 2013 | 2009 - 2010  (14 months) | Calabar, Nigeria | Prospective, unspecified enrolment. Pattern of respiratory mycoses in HIV. | Tertiary hospital ARV clinics | Respiratory tract infection and producing sputum. 331 enrolled, 272 HIV+. | Expectorated sputum: Grocott | Mean 342 | NS | NS | Uncertain | 9 |
| Van Oosterhout 2007 | 2002 - 2004  (24 months) | Blantyre, Malawi | Prospective cohort study of incidence of cotrimoxazole-preventable infections in HIV. Incidence of PCP in the cohort. | Primary care clinic | Suspected PCP on clinical grounds. Cohort size 660; 95 episodes of suspected PCP in 75 patients. | Induced sputum: IFA and PCR. Clinical case definition | CD4 < 200 in 284 patients | None | None | Low | 10 |

**In- and outpatients**

| **Study** | **Period** | **Location** | **Design and objective** | **Setting** | **Participants** | **Diagnosis** | **CD4 count** | **ART** | **PCP prophylaxis** | **Bias** | **Quality score** |
| --- | --- | --- | --- | --- | --- | --- | --- | --- | --- | --- | --- |
| Aderaye 2007 | 2004 - 2005  (16 months) | Addis Ababa, Ethiopia | Prospective, consecutive. Prevalence of PCP and other pulmonary infections in HIV with sputum AFB -ve. | Tertiary hospital | All patients with respiratory symptoms, atypical CXR and sputum AFB -ve. 131 enrolled. BAL if sputum IFA -ve or no sputum available. | Expectorated sputum (n = 78) and BAL (n = 118): IFA only. Clinical case definition. | Median 82 | None (new HIV) | 1/131 | Uncertain | 11 |
| Hargreaves 2001 | 1997 - 1998  (8 months) | Lilongwe, Malawi | Prospective, consecutive. Prevalence and outcomes of PCP in patients with smear -ve TB. | Central hospital | All patients with suspected smear -ve TB. 352 enrolled (278 HIV+); 253 eligible and 193 underwent bronchoscopy, BAL in 186 (164 HIV+). | BAL: IFA and PCR. Sputum not tested | NS | NS | NS | High | 9 |
| Lockman 2003 | 1997  (12 months) | Gabarone and Francistown Botswana | Prospective cohort study. Prevalence and outcomes of suspected PTB and other causes of cough. | District hospitals | Cough or constitutional symptoms > weeks. 229 enrolled: 32 outpatients, 197 HIV+, 121 tested for PCP. Only 45 had RR > 25. 10% too ill to enrol or died before enrolment. | Induced sputum: TBO or MS and PCR | Median 73 | NS | NS | High | 10 |
| Nowaseb 2014 | 2011 (3 months) | Windhoek, Namibia | Prospective, laboratory-based, consecutive specimens.  Performance of expectorated sputum for PCP daignosis. | Tertiary hospital | All sputum submitted for TB microscopy, adults > 16 years old, unclear symptoms. 475 sputum samples, 175 from HIV+. | Expectorated sputum: GMS and PCR | Median  282 | NS | NS | High | 8 |
| Okwera 2013 | 2008 - 2011  (3 years) | Kampala, Uganda | Prospective, consecutive. Prevalence of PCP and other pulmonary infections in HIV+ with recurrent episode of suspected smear -ve TB. | Tertiary hospital | HIV+, previous TB with cough and respiratory symptoms > 2 weeks with sputum AFB -ve. Chronic illness and Karnofsky performance < 50% excluded. 634 screened, 178 enrolled. | Induced sputum: PCR (unclear if also used Giemsa) | Median 260.5 | 84/178 | 156/178 | High | 10 |

**Post-mortem studies**

| **Study** | **Period** | **Location** | **Design and objective** | **Setting** | **Participants** | **Diagnosis** | **CD4 count** | **ART** | **PCP prophylaxis** | **Bias** | **Quality score** |
| --- | --- | --- | --- | --- | --- | --- | --- | --- | --- | --- | --- |
| Ansari 2002 | 1997 - 1998  (12 months) | Francistown, Botswana | Prospective autopsy study of consecutive medical deaths. Causes of death in HIV+ patients. | Inpatients: referral centre | All non-trauma deaths. Prioritised: no diagnosis, unexplained deterioration, respiratory illness. 5055 admissions, 565 HIV-associated deaths; 104 HIV+ autopsies. | Histology: Grocott staining | NS | NS | NS | High | 10 |
| Bates 2015 | 2012 - 2013 (13 months) | Lusaka, Zambia | Prospective consecutive autopsy study. Diseases at PM stratified by HIV status. | Inpatients: tertiary hospital | All inpatient deaths in medical ward. 1372 deaths, 125 autopsies, 101 HIV-infected. | Histology: Grocott staining | NS | 48/96 | NS | High | 10 |
| Cox 2012 | 2009  (5 months) | Kampala, Uganda | Prospective consecutive autopsy study. Causes of death in infectious diseases and gastroenterology wards. | Inpatients: tertiary hospital | All weekday deaths with consent. 290 deaths, 59 autopsies analysed, 35 HIV+. | Histology: Grocott staining | Mean 50 | 10/35 | 28/35 | High | 10 |
| Cox 2014 | 2013  (5 months) | Kampala, Uganda | Prospective consecutive autopsy study. Accuracy of needle biospy to determine cause of death in HIV. | Inpatients: tertiary hospital | All HIV+ deaths in medical wards.  99 enrolled (total deaths not reported), 96 underwent PM. | Histology: Grocott staining | 47 | 59/96 | 75/96 | Uncertain | 10 |
| Domoua 1995 | 1989 - 1990  (9 months) | Abidjan, Cote d' Iviore | Prospective consecutive autopsy study. Causes of death in HIV+ patients with respiratory symptoms. | Inpatients: central hospital | All HIV+ patients with respiratory illness atypical of TB. 70 included and underwent PM. | Histology: Grocott staining | NS | NS | NS | Low | 10 |
| Martinson 2007 | 2003 - 2005  (15 months) | Soweto, South Africa | Prospective autopsy study and retrospective folder review. Causes of death in HIV+ patients on TB treatment. | Inpatients: central hospital | Premortem diagnosis of TB, death after 24 hours of admission. 1000 eligible deaths, PM performed on 47 HIV+ patients. | Histology: Grocott staining and PCR | Mean 48 | None | 18/47 | High | 10 |
| Menendez 2008 | 2002 - 2004  (26 months) | Maputo, Mozambique | Prospective consecutive autopsy study to determine causes of maternal death. | In and outpatients | All maternal deaths. 139/179 deaths included in analysis. 65 HIV+ (123 women tested for HIV). | Histology: unspecified staining | NS | NS | NS | High | 10 |
| Murray 2007 | 1990s - 2002 (duration NS) | South Africa | Retrospective autopsy and clinical review of gold miner cohort. Rates and causes of death in miners. | In and outpatients | Analysis of all HIV-related deaths. 242 HIV+ deaths from natural causes; PM performed in 66. | Histology: Grocott staining | NS | None | NS | High | 9 |
| Rana 2000 | 1996 - 1997 (11 months) | Nairobi, Kenya | Prospective consecutive autopsy study. Causes of inpatient deaths. | Inpatients: tertiary hospital | All medical deaths consenting to autopsy. 155 HIV+ deaths, 75 underwent PM. | Histology: Grocott staining | Median 160 (of random sample) | NS | NS | High | 10 |
| Rana 1997 | 1995  (10 months) | Nairobi, Kenya | Prospective consecutive autopsy study. Determine major pathology in HIV+ inpatient deaths. | Inpatients: tertiary hospital | HIV+ deaths in study of isoniazid preventive therapy. PM performed in 9 patients; no description of study population or total deaths. | Histology: unspecified staining | Median 63 | NS | NS | High | 10 |
| Wong 2012 | 2009  (12 months) | Johannesburg, South Africa | Prospective consecutive autopsy study. Causes of death in HIV+ inpatients on ART. | Inpatients: tertiary hospital | HIV+ inpatients on ART or eligible for ART. 39 enrolled and underwent PM. | Histology: Grocott staining | Median 50 | 25/39 | 14/39 | Low | 10 |

**Case fatality studies**

| **Study** | **Period** | **Location** | **Design and objective** | **Setting** | **Participants** | **Diagnosis** | **CD4 count** | **ART** | **PCP prophylaxis** | **Bias** | **Quality score** |
| --- | --- | --- | --- | --- | --- | --- | --- | --- | --- | --- | --- |
| Karstaedt 2001 | 1996 - 1998 (36 months) | Soweto, South Africa | Retrospective record review. Description of clinical and laboratory features of PCP. | Inpatients: tertiary hospital | 120 confirmed PCP cases (0.6% of all HIV+ admissions) | Induced sputum: IFA in 65. Expectorated sputum: IFA in 50, BAL in 5 | NS | NS | NS | Uncertain | NA |
| Orlowic 2001 | 1997 - 1999 (22 months) | Johannesburg, South Africa | Retrospective case series. Features of PCP/TB coinfection. | Inpatients: tertiary hospital | Inpatients with PCP and TB. 39 cases of dual infection. | Induced sputum: IFA | NS | NS | NS | Uncertain | NA |

Abbreviations: CXR chest X-ray; AFB acid-fast bacilli; OI opportunistic infection; TB tuberculosis; BAL bronchoalveolar lavage; IFA immunofluorescent antibody test; NS not specified; TBB transbronchial biopsy; -ve negative; +ve positive; PCR polymerase chain reaction; TBO toluidine blue O; ART antiretroviral therapy; PM post-mortem, GMS Gomori methanamine silver, MS methanamine silver.

Quality scores: good, 11; moderate, 10; poor ≤ 9
