# Supplementary material for: Burden of pneumocystis pneumonia in HIV-infected adults in sub-Saharan Africa: a systematic review and meta-analysis
Source: BMC Infect Dis. 2016 Sep 9;16(1):482. doi: 10.1186/s12879-016-1809-3 (PMC5018169; doi:10.1186/s12879-016-1809-3)
Supplement: Additional file 4: — Figure showing bias and quality assessment. (PPTX 93 kb) [file 12879_2016_1809_MOESM4_ESM.pptx]

## Slide 1
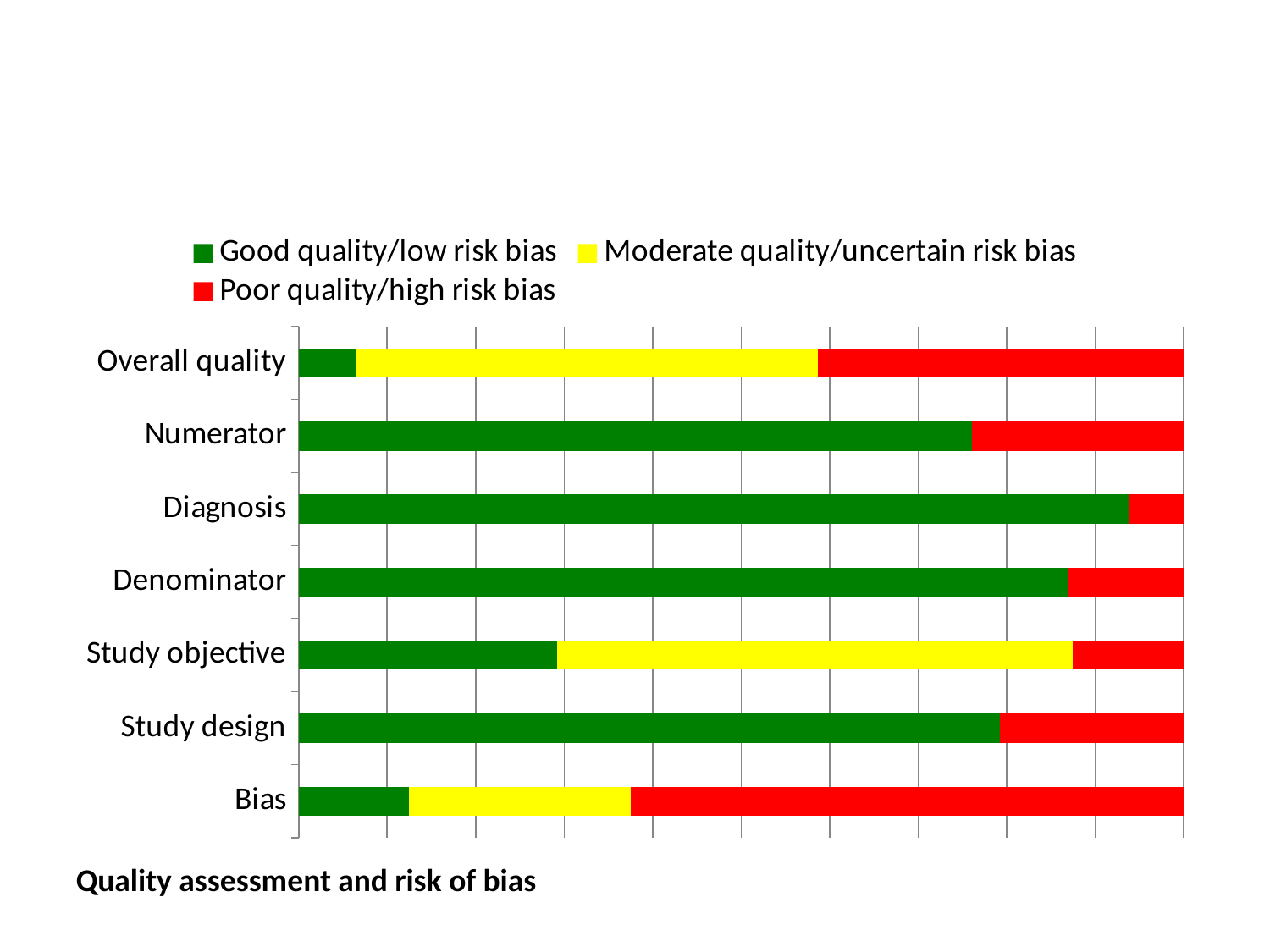

### Chart
| Category | Good quality/low risk bias | Moderate quality/uncertain risk bias | Poor quality/high risk bias |
|---|---|---|---|
| Bias | 6.0 | 12.0 | 30.0 |
| Study design | 42.0 | 0.0 | 11.0 |
| Study objective | 14.0 | 28.0 | 6.0 |
| Denominator | 40.0 | 0.0 | 6.0 |
| Diagnosis | 45.0 | 0.0 | 3.0 |
| Numerator | 35.0 | 0.0 | 11.0 |
| Overall quality | 3.0 | 24.0 | 19.0 |Quality assessment and risk of bias
